# Measuring Quality of Mental Health Care: An International Comparison

**DOI:** 10.3390/ijerph111010384

**Published:** 2014-10-10

**Authors:** Brigitta Spaeth-Rublee, Harold Alan Pincus, Fran Silvestri, Janet Peters

**Affiliations:** 1 Department of Psychiatry, Columbia University, New York, NY 10032, USA; E-Mail: bspaeth@nyspi.columbia.edu; 2 Irving Institute for Clinical and Translational Research, New York, NY 10032, USA; 3 NewYork-Presbyterian Hospital; New York, NY 10032, USA; 4 The International Initiative for Mental Health Leadership, Millen Ave, Pakuranga 2010, New Zealand; E-Mails: fran@iimhl.com (F.S.); janet@iimhl.com (J.P.)

The International Initiative for Mental Health Leadership (IIMHL) (www.iimhl.com) is a unique international collaborative that focuses on improving mental health and addiction services. IIMHL is a collaboration of eight countries including Australia, England, Canada, New Zealand, Republic of Ireland, Scotland, Sweden and USA.

Effective leadership will promote better outcomes for people who use mental health and addiction services and their families. IIMHL organizes systems for international networking, innovation sharing and problem solving across countries and agencies. One of IIMHL’s best recognized initiatives for linking leaders internationally is the ‘Leadership Exchange’. The Leadership Exchange is a week-long learning event which is held every 16 months. Knowledge transfer through IIMHL includes not only the Leadership Exchange, but also promotion of workshops/training/education, support of learning collaboratives, research and information dissemination between Exchanges. Joining IIMHL is free to any leader however the Leadership Exchange is only free to leaders from one of the eight participating countries.

The project, “Measuring Quality of Mental Health Care: An International Comparison”, was initiated by a group of clinical experts under the auspices of the IIMHL Clinical Leaders Group. Led by Prof. Harold Pincus from Columbia University in New York, the project aims to not only raise awareness among clinicians and policymakers regarding the quality of care of the mental health systems they are working in, but ultimately to be able to compare system performance across countries to inform initiatives for transformation of mental health services. In addition to the eight core countries, outreach was made to mental health experts in Germany, Japan, Taiwan, the Netherlands, and Norway and consequently, these countries participated in various stages of the three-phase project as well. The three-phase project uses an inductive approach by deriving examples of domains and quality measures through a compilation of existing measurement programs, thus generating a broad and maximally inclusive list of potentially collectable measures across participating countries.

This paper provides a brief overview of the activities undertaken in Phases I, II and III as well as a brief summary of research highlights from papers that have come out of this project so far.

## 1. Phase I

For Phase I of the project, we collected information from the participating IIMHL countries and others (Australia, Canada, England, Germany, Ireland, Japan, The Netherlands, New Zealand, Norway, Scotland, Taiwan, and the United States) by conducting an international literature review (including a review of grey literature such as government reports, white papers and others) of population-based performance measurement initiatives in mental health. The review revealed 55 studies and reports from 12 countries at the national and provincial level and three cross-national initiatives by international organizations such as the Organization for Economic Cooperation and Development (OECD), the World Health Organization (WHO), and the European Union (EU) which reported on a wide range of quality measures and domains [1]. This initial phase of the three-phase study aimed at providing an overview and assessment of the broader context of mental health quality measurement in each of the participating countries. These activities also resulted in a number of additional publications by country leaders which provided a snapshot of international efforts to measure and improve the quality of mental health care within their respective countries [2,3,4,5,6,7].

The wide array of quality domains identified through the international literature review may be indicative of specific policy agendas that reflect not only differences in the structure and organization of health care systems, but the unique situation of mental health care provision and delivery within these broader systems as well as the data sources available.

As part of Phase I of the project, we also developed and implemented a survey instrument to identify and assess ongoing or soon to-be-established national-level mental health quality measurement programs in the 12 participating countries. The aim was to gain a better understanding of the nature and structure of these programs as well as the overall mental health system in which these programs operate. The survey identified thirty-eight programs, which were mostly administered by governmental organizations (66%), focused on hospital care (76%), and used encounter or utilization databases as sources of information (61%). Reflecting some of the findings of the international literature review, the survey found that programs and program data were used for various purposes and presented a wide range of domains of quality. Most commonality was seen in domains associated with high-acuity care while relatively few programs were assessing recovery-related domains [8].

The international literature review yielded a total of 656 indicators which were classified and assigned to a list of 17 domains and 80 sub-domains adapted from the National Inventory of Mental Health Quality Measures. By collecting and organizing measures through an inductive compilation of existing programs, we were able to generate a maximally inclusive basis. This coding process laid the groundwork for further refinement of the indicator list in Phase II. The number of indicators per domain and subdomain were highly variable; however, no single program contained indicators in all domains [9].

## 2. Phase II

The focus of Phase II of the project was to develop consensus for a core set of performance and outcome measures that could be potentially collected by all participating countries.

Part of this process was to filter and narrow down the overall number of 656 indicators collected in Phase I. In a multi-phased process, this list was narrowed down to 36 measurement concepts (and associated base indicators) and 10 domains: In addition to eliminating measures due to duplication, incomplete indicator or data information, measures with minor variations in numerator and denominator definitions were combined into *measurement concepts* (range of indicators which use minor modifications of numerator and/ or denominator definitions to measure similar processes or outcomes in mental health care)*.* Most of the 36 associated based indicators were directly derived from the original list of 656 indicators and thus present a compendium of measures that are actually in use to measure quality in (some) participating countries. In some instances, however, “generic” base indicators were developed and proposed for rating to better reflect the range of information available to us through the composite of international mental health indicators and potentially facilitate adaptation by other countries.

### Delphi Process

Applying a modified Delphi process, we conducted multiple rounds of surveys where we asked country experts to rate 36 measurement concepts and 36 associated base indicators derived from the list of 656 indicators collected in Phase I according to their validity, importance, and feasibility. A measure was considered important if it captured a difference between actual and potential level of care, addressed an issue of concern to policymakers and consumers, and applied to a significant share of the population. A measure was regarded feasible if data could be collected either in most/all of any of the participating countries or by some subgroups such as specific regions, provider groups or organizations.

A 2-day meeting in September in New York City in 2012 provided 14 experts from 9 countries with the opportunity to discuss the preliminary survey results. Among the 10 most highly ranked indicators for validity as well as importance were seclusion, 7-day follow-up after inpatient discharge, medication errors, restraint, death rates, medication adherence and involuntary/compulsory hospitalization. Seclusion and restraint, which were among the three most highly rated measurement concepts for validity were also among the top three rated indicators for importance.

Patient involvement, symptom reduction and functioning were among the ten most highly ranked measurement concepts for importance but are not represented among the most highly ranked concepts for validity. Likewise, screening for substance use disorders, continuity of care plan/ discharge plan, and polypharmacy while being among the top-10 ranked measurement concepts for validity received lower rating for importance and are not among the top-rated ones [10]. The Delphi method applied here is not without limitations: the expert group consensus may reflect the judgment of a select group of people and as such may have limited representation. In addition, this method does not reflect a quantitative assessment of evidence.

In addition, a paper on alternative operationalizations of quality measures based on the results of the Delphi process is in development.

## 3. Phase III

Based on the work previously achieved in Phase I and II, Phase III is focusing on pilot efforts to identify and collect data on a selected number of indicators across participating countries (Australia, Canada, England, Ireland, New Zealand, Germany, the Netherlands, Norway, Scotland, and the US).

Responding to a previously identified gap with regard to recovery measures and domains as demonstrated in the results of the international literature review and survey conducted in Phase I, we are also seeking to develop a limited set of recovery oriented measures for quality improvement and accountability which could be implemented and collected across participating countries. Another gap identified through our ongoing discussions pertains to the assessment of the quality of care for patients within the criminal justice system and the importance to evaluate if and how any of the existing measures can be adapted or if new quality measures need to be identified and developed.

### Survey

We developed and implemented a survey to review the feasibility of the most highly ranked indicators in the Delphi process of Phase II of the project, review current definitions of those indicators across participating countries, and include additional indicators for which countries are currently collecting data for further consideration. The results of the survey were discussed during a 2-day meeting of the IIMHL Clinical Leaders Group in England (June 2014). Experts from 8 countries (Australia, Canada, England, the Netherlands, Norway, New Zealand, Scotland, and the United States) reviewed the survey results and provided additional input and clarification on indicator definitions and data availability. Particular focus was put on the next phase in gathering data, identifying other important domains not covered by this survey such as recovery and how the data collection process can be further validated. We will continue to review and refine the responses to the measure survey including data collection in close collaboration with country experts. A full summary report of findings of Phase III will be delivered to country representatives by the end of 2014.

In addition, we have been conducting an international literature review (review of reviews) of existing recovery oriented measures and compilation of identified measures. Recommendations on how to move forward on a reconciled and commonly agreed upon list of recovery measures will be part of a separate report due by the end of this year.

## 4. Outlook

Going forward, this international initiative is seeking to create more effective, valid and feasible methods for measuring the quality of mental health, share information and experiences and collaborate with other organizations and networks toward improving the delivery of mental health care and ultimately comparing and benchmarking health care services across countries. Potential future work in this area might involve collaboration among participating countries to focus on selected indicators for which there is extensive variation and room for improvement within their national contexts.

One important aspect to continue this effort beyond the duration of this project is the creation of an international support network. To this end, we initiated a process to create or link internal interest groups and networks related to quality measurement in each country. These networks, consisting of IIMHL clinical lead members and other designated experts, have been providing input/ feedback on the development process of the framework and served as respondents to the various surveys in Phase I-III. These experts are also helping to facilitate the implementation and data collection on a selected number of indicators for performance measurement and improvement of mental health within their respective countries and to advance the further development and refinement of core indicators to be collected in participating countries beyond the duration of this project. In addition, we are in regular contact with experts at the OECD and WHO to explore avenues of collaboration and coordination.

While there are clearly major challenges in standardizing quality measurement across countries, given the large differences in structures, policies and funding of health and mental health systems, there is significant agreement on the importance of quality measurement as well as identifying and prioritizing measurement concepts that could be implemented across participating countries. There is much that can be learned by sharing strategies, efforts and experiences in individual countries to meet these challenges.
